# A Prospective Cohort Study Examining the Associations of Dietary Calcium Intake with All-Cause and Cardiovascular Mortality in Older Chinese Community-Dwelling People

**DOI:** 10.1371/journal.pone.0080895

**Published:** 2013-11-05

**Authors:** Ruth Chan, Jason Leung, Jean Woo

**Affiliations:** 1 Department of Medicine and Therapeutics, The Chinese University of Hong Kong, Hong Kong SAR; 2 Jockey Club Centre for Osteoporosis Care and Control, The Chinese University of Hong Kong, Hong Kong SAR; Kurume University School of Medicine, Japan

## Abstract

**Background:**

Most epidemiological studies of calcium intake and mortality risk have been conducted in populations with moderate to high calcium intake, and limited studies have focused on populations with low habitual calcium intake (i.e., mean dietary calcium intake <700 mg/d).

**Objective:**

This study investigated the association between dietary calcium intake and death from all causes and cardiovascular disease in Chinese population with low habitual calcium intake.

**Design:**

Data from 3,139 Chinese men and women in a population-based prospective cohort study, aged >=65 years and free of heart diseases or stroke at baseline, were analyzed. Primary outcome measures, identified from the death registry, were death from all causes and cardiovascular disease. Dietary calcium intake assessed using a validated food frequency questionnaire was categorized into sex-specific quartiles. Data on use of supplemental calcium (yes or no) including individual calcium supplements and other calcium containing supplement were collected. Cox regression models adjusted for demographic and lifestyle variables were used to estimate hazard ratios (HRs) and 95% confidence intervals (CI).

**Results:**

During a median of 9.1 years of follow-up, 529 all-cause deaths (344 men, 185 women) and 114 (74 men, 40 women) deaths from cardiovascular disease were identified. An inverse trend between dietary calcium intake and mortality was observed. Compared with the lowest quartile (<458 mg/d for men, <417 mg/d for women), the highest quartile of dietary calcium intake (>762 mg/d for men, >688 mg/d for women) had a significantly reduced risk of all-cause mortality (multivariate HR=0.63, 95% CI=0.49-0.81, *P*_trend_<0.001) but an insignificant decreased risk of cardiovascular mortality (multivariate HR=0.70, 95% CI=0.41-1.21, *P*_trend_=0.228). Similar inverse association was observed when the analyses were stratified on calcium supplemental use.

**Conclusions:**

Higher intake of dietary calcium was associated with reduced risk of all-cause mortality and possibly cardiovascular mortality in Chinese older people with low habitual calcium intake.

## Introduction

Increasing calcium intake has been encouraged because of its beneficial effect on bone health. Recent evidence has however raised concern about the potential adverse effect of high calcium intake, in particular the intake of supplemental calcium on cardiovascular health [1]. The majority of the epidemiological studies examining the association between calcium intake and cardiovascular health have been conducted in White population with moderate to high calcium intake. Among these studies, only a few of them have examined calcium intake in relation to cardiovascular mortality as the study outcome [2]. 

In the National Institutes of Health-American Association of Retired Persons (AARP) Diet and Health Study, supplemental calcium intake of more than 1,000 mg/day or total calcium intake of 1,500 mg/day and higher was associated with elevated total cardiovascular disease (CVD) mortality in men but not in women, whereas dietary calcium intake was not related to total CVD death in either sex [3]. In contrast, elevated all-cause and CVD mortality was observed among women with dietary calcium intake exceeding 1,400 mg/day compared with women with lower intakes in a female cohort study from Sweden. Of particular interest were the observations that the use of calcium tablets was on average not associated with all-cause or cause specific morality, but supplemental calcium use further increased risk of death among women with dietary calcium intake above 1,400 mg/day in a dose dependent manner [4]. 

The findings of these studies therefore suggest that elevating calcium intake regardless of the sources increases mortality, in particular CVD mortality. However, the relationship is possibly dependent on habitual calcium intake. High calcium intake may have a protective effect on mortality in populations, such as Chinese with comparatively low habitual calcium intake [5,6]. To our knowledge, no study has examined the association between dietary calcium intake and all-cause and CVD mortality in Chinese population. This study therefore aimed to provide findings regarding this aspect, using data from a sample of older Chinese people in Hong Kong with low habitual calcium intake (i.e. mean dietary calcium intake less than 700 mg/d) [5,7]. We expected that increasing dietary calcium intake would be associated with reduced mortality risk in our sample with low habitual calcium intake.

Results of the present study were built on from our preliminary findings that an inverse trend between dietary calcium intake and mortality was observed in a cohort of Chinese older people with comparatively low habitual calcium intake [8]. The present study was different from the preliminary analyses in several aspects. The preliminary analyses examined the association between calcium intake and mortality with focus on dietary calcium intake only and the multivariate models were only controlled for few demographic and lifestyle factors. In contrast, the present study added more results of the stratified analysis by supplemental calcium use, and included more potential covariates, such as self-reported history of diabetes and hypertension and macronutrient intakes in the multivariate analyses.

## Subjects and Methods

### Study population

This study was conducted in accordance with the Declaration of Helsinki. This study was approved by the Clinical Research Ethics Committee of the Chinese University of Hong Kong. Written informed consent was obtained from all participants. Subjects were participants of a prospective cohort study examining the risk factors for osteoporosis in Hong Kong [9]. Four thousand Chinese men (n=2,000) and women (n=2,000) aged 65 years and over living in the community were recruited between 2001 and 2003 by recruitment notices and talks in community centers and housing estates. Participants were volunteers and were able to walk or take public transport to the study site. .

We excluded participants who fell into one of the following categories for analysis: those with a history of heart diseases or stroke at baseline, those without dietary data, and those with extreme daily energy intake at the 0.5^th^ and 99.5^th^ percentiles of the sex-specific range. A final sample of 3,139 participants was used for data analysis. 

### Demographic and overall health characteristics

A standardized interview was performed to collect information on age, gender, education level, smoking habit, alcohol use, calcium supplemental use and medical history. Information on the duration and level of past and current use of cigarettes, cigars and pipes was obtained. Smoking history was classified in terms of former smoking (at least 100 cigarettes smoked in a lifetime), current smoking and never smoking. Drinking status was defined as never, former (ever drank at least 5 drinks daily in a lifetime) or current drinker. Self-reported calcium supplemental use including use of individual calcium supplements or other types of calcium containing supplements was also asked, however data on the frequency and dosage of calcium supplemental use were not collected in the present study. Therefore, the calcium supplemental use was coded as a dichotomous variable (yes or no) in the present study. Baseline disease status was obtained by self-report of their doctors’ diagnoses, supplemented by the identification of drugs brought to the interviewers. 

### Anthropometric data

Body weight was measured to the nearest 0.1 kg with participants wearing a light gown, using the Physician Balance Beam Scale (Healthometer, Illinois, USA). Height was measured to the nearest 0.1 cm using the Holtain Harpenden stadiometer (Holtain Ltd, Crosswell, UK). Body mass index (BMI) was calculated as body weight in kg / (height in m)^2^.

### Physical activity assessment

Physical activity was assessed by the Physical Activity Scale for the Elderly (PASE) [10]. This is a 12-item scale measuring the average number of hours per day spent in leisure, household, and occupational physical activities over the previous 7-day period. Activity weights for each item were determined based on the amount of energy spent, and each item score was calculated by multiplying the activity weight with daily activity frequency. A composite PASE score of all the items was yielded. A higher PASE score reflects higher physical activity level.

### Dietary assessment

Baseline dietary intake was assessed using a validated food frequency questionnaire (FFQ) developed in a population survey [11]. Daily nutrient intake from diet was calculated using food tables derived from McCance and Widdowson [12] and the Chinese Medical Sciences Institute [13]. The FFQ consisted of 280 food items. Each participant reported the food item, the size of each portion, the number of times of consumption each day and each week, using the past 12 months prior to the interview as a reference period. Portion size was explained to participants using a catalogue of pictures of individual food portions. Dietary nutrient intakes were adjusted for total energy intake using the residual method for data analysis [14].

### Mortality ascertainment

Data on all-cause and CVD mortality were obtained from Death Registry of the Department of Health of the Hong Kong SAR Government where all deaths in Hong Kong are registered. Cause of death was determined by review of death certificates and was coded using the International Classification of Diseases-version 10 (ICD-10). CVD mortality referred to cause of death with ICD-10 code of I00-I99. These data were checked by four-monthly telephone interviews conducted by trained research assistants who called each individual’s family members, and data on mortality statistics were collected through March 2012 with a median follow-up of 9.1 years.

### Statistical analysis

Statistical analyses were performed using the statistical package SPSS version 17.0 (SPSS Inc., Illinois, US). Data was checked for normality by descriptive analysis. Student’s *t* test and chi square test were used to examine the baseline differences in mean age, BMI, PASE, dietary intake of energy and other nutrients, and also the differences in the distribution of education level, smoking habit, alcohol use, calcium supplemental use, and self-reported history of diabetes and hypertension between participants included and participants excluded for analysis, and between those who died and those who were alive at the follow-up.

Dietary energy adjusted calcium intake was stratified into sex-specific quartiles based on the distribution of the included sample for analysis. Interaction between sex and quartiles of dietary energy adjusted calcium intake was tested by addition of cross-product terms to the multivariate models. Interactions were not significant, thus all analyses are presented in the total population. Using Cox proportional hazards regression we estimated age and sex adjusted, and multivariable adjusted hazard ratios (HRs) and their 95% confidence intervals (CIs) for all-cause mortality or CVD mortality during the follow-up period according to continuous dietary energy adjusted calcium intake or quartiles of dietary energy adjusted calcium intake. The follow-up period was defined as the time from the baseline examination to the date of death (for all-cause mortality analysis) / death from CVD (for CVD mortality analysis), or date to the latest database update (i.e. March 2012), whichever came first. Multivariate models were adjusted for various baseline parameters, including age, BMI, PASE, daily energy intake, percentage of energy from total fat and saturated fat (all continuous), sex (male or female), education level (primary or below, secondary, or University and above), smoking status (never, former or current), alcohol use (never, former or current), calcium supplemental use (yes or no) and self-reported history of hypertension and diabetes (yes or no). These parameters were selected based on the fact that there were significant differences in these parameters between those who died and those who were alive, and past studies supporting that the parameters were considered as potential risk factors for mortality [15]. The assumption of the Cox proportional hazards models was validated by parallel curves of log-minus-log plots. Test for trend was examined by entering quartiles of energy adjusted calcium intake as a fixed factor and testing the contrast by using the polynomial option in all models. An α level of 5% was used as the level of significance.

## Results

Participants who were excluded for analysis were older, more likely to be male, non-drinkers, and more likely to have self-reported history of diabetes and hypertension than participants who were included for analysis. There was no significant difference in dietary calcium intake between the two groups (data not shown). 

During a median of 9.1 years of follow-up, 529 all-cause deaths (344 men, 185 women) and 114 CVD deaths (74 men, 40 women) were identified. Mean (SD) and median (IQR) of raw dietary calcium intake of the studied population was 596.1 (279.4) mg/d and 545.3 (393.5-741.0) mg/d respectively. Baseline characteristics of the final sample by mortality status are shown in Table 1. Participants who died were generally older, less physically active, had lower dietary calcium intake, more likely to be male, smokers, and calcium supplement nonusers, and more likely to have self-reported history of diabetes and hypertension than participants who were alive.

**Table 1:** Selected baseline characteristics of study participants by mortality status.

Baseline characteristics	All-cause mortality (n=3.139)		CVD mortality (n=3,139)	
	Death (All) (n=529)	Alive (n=2,610)	Death (CVD) (n=114)	Alive or death from other causes (n=3,025)
Age (y)	75.3 (6.0)	71.8 (4.8)	76.0 (5.7)	72.3 (5.1)
BMI (kg/m^2^)	23.0 (3.6)	23.6 (3.2)	23.2 (3.7)	23.5 (3.2)
PASE	86.4 (43.3)	93.0 (43.1)	82.6 (41.1)	92.3 (43.2)
Male (%)	65.0	45.6	64.9	48.3
Energy intake (kcal)	1823.5 (550.0)	1833.7 (565.6)	1769.7 (581.7)	1834.3 (562.2)
Energy from total fat (%)	28.5 (6.2)	28.2 (6.3)	29.0 (5.8)	28.2 (6.3)
Energy from saturated fat (%)	6.6 (1.9)	6.5 (1.9)	6.6 (1.8)	6.5 (1.9)
Energy adjusted calcium intake (mg)^1^	570.3 (227.1)	601.2 (229.4)	588.8 (212.0)	596.3(230.0)
Q1 (%)	32.1	23.6	28.1	24.9
Q2 (%)	25.1	25.0	24.6	25.0
Q3 (%)	21.7	25.6	24.6	25.0
Q4 (%)	21.0	25.8	22.8	25.1
Smoking status				
Never (%)	46.1	67.8	52.6	64.6
Former (%)	42.9	25.6	39.5	28.1
Current (%)	11.0	6.7	7.9	7.4
Alcohol use^2^				
Never (%)	81.1	85.2	81.6	84.6
Former (%)	4.2	1.2	5.3	1.5
Current (%)	14.7	13.7	13.2	13.9
Education				
Primary or below (%)	76.0	70.8	74.6	71.5
Secondary (%)	17.0	19.0	14.0	18.9
University or above (%)	7.0	10.2	11.4	9.6
Self-reported history				
Diabetes (%)	18.1	11.8	21.9	12.5
Hypertension (%)	44.6	36.6	50.0	37.5
Calcium supplemental use (%)	9.8	14.7	7.9	14.1

^1^ Adjusted for mean total energy intake (2096 kcal for men, 1579 for women) using the residual method. The quartiles values of energy adjusted calcium intake were <458, >458 to <584, >584 to <762 and >762 mg/day for men, and <417, >417 to <529, >529 to <688 and >688 mg/day for women.

^2^ 2 participants with missing data

We stratified the whole participants according to the sex-specific quartiles of dietary energy adjusted calcium intake. An inverse trend between dietary calcium intake and all-cause or CVD mortality was observed (Table 2). Compared with the lowest quartile (<458 mg/d for men, <417 mg/d for women), the highest quartile of dietary calcium intake (>762 mg/d for men, >688 mg/d for women) had a significantly reduced risk of all-cause mortality (HR=0.63, 95% CI=0.49-0.81, *P*_trend_<0.001) and an insignificant decreased risk of CVD mortality (HR=0.70, 95% CI=0.41-1.21, *P*_trend_=0.228) in the age and sex adjusted models. Similar results were observed when the models were further adjusted for BMI, education level, self-reported history of diabetes and hypertension, calcium supplemental use, and other lifestyle factors. Table 3 shows the Cox proportional hazards regression results stratified on calcium supplemental use. The risk estimates for all-cause mortality according to quartiles of energy adjusted calcium intake were similar in the two strata. However, there was a larger discrepancy in the risk estimates for CVD mortality in the two strata, possibly due to the few number of CVD deaths among calcium supplemental users in the present study. 

**Table 2:** HRs and 95% CIs for all-cause and CVD mortality by quartiles of energy adjusted dietary calcium intake.

	Energy adjusted calcium intake^1^	No. of deaths	No. of person-years	Rate per 1,000 person years	Crude		Model 1^2^		Model 2^3^	
					HR	95% CI	HR	95% CI	HR	95% CI
Mortality	Continuous (per SD)	529	27,288	19.4	0.88	0.80-0.96	0.84	0.77-0.92	0.84	0.76-0.92
(all-cause)	*P*				0.004		<0.001		<0.001	
	Q1	170	6,735	25.2	1 (ref)		1 (ref)		1 (ref)	
	Q2	133	6,772	19.6	0.78	0.62-0.98	0.79	0.63-0.99	0.73	0.58-0.92
	Q3	115	6,896	16.7	0.66	0.52-0.84	0.67	0.53-0.85	0.65	0.51-0.83
	Q4	111	6,886	16.1	0.64	0.50-0.81	0.64	0.51-0.82	0.63	0.49-0.81
	*P_trend_*				<0.001		<0.001		<0.001	
Mortality	Continuous (per SD)	114	27,288	4.2	0.96	0.79-1.15	0.92	0.77-1.11	0.91	0.74-1.12
(CVD)	*P*				0.639		0.403		0.387	
	Q1	32	6,735	4.8	1 (ref)		1 (ref)		1 (ref)	
	Q2	28	6,772	4.1	0.88	0.53-1.46	0.89	0.53-1.47	0.77	0.46-1.30
	Q3	28	6,896	4.1	0.86	0.52-1.43	0.88	0.53-1.46	0.78	0.46-1.33
	Q4	26	6,886	3.8	0.81	0.48-1.35	0.82	0.49-1.37	0.75	0.44-1.30
	*P_trend_*				0.418		0.454		0.346	

^1^ Adjusted for mean total energy intake (2096 kcal for men, 1579 for women) using the residual method. The quartiles values of energy adjusted calcium intake were <458, >458 to <584, >584 to <762 and >762 mg/day for men, and <417, >417 to <529, >529 to <688 and >688 mg/day for women.

^2^ Adjusted for age and sex

^3^ Further adjusted for BMI, PASE, smoking status, alcohol use, education level, self-reported history of diabetes and hypertension, energy intake, percentage of energy from total fat, percentage of energy from saturated fat, and calcium supplemental use

**Table 3:** HRs and 95% CIs for all-cause and CVD mortality by quartiles of energy adjusted dietary calcium intake stratified by calcium supplemental use.

	Calcium supplemental use	Energy adjusted calcium intake^1^	No. of deaths	No. of person- years	Rate per 1,000 person years	Crude		Model 1^2^		Model 2^3^	
						HR	95% CI	HR	95% CI	HR	95% CI
Mortality	No	Continuous (per SD)	477	23,477	20.3	0.87	0.79-0.96	0.83	0.76-0.92	0.82	0.74-0.91
(all-cause)		*P*				0.004		<0.001		<0.001	
		Q1	156	5,935	26.3	1 (ref)		1 (ref)		1 (ref)	
		Q2	120	5,938	20.2	0.77	0.61-0.98	0.78	0.62-1.00	0.72	0.57-0.93
		Q3	102	5,800	17.6	0.67	0.52-0.86	0.68	0.53-0.87	0.64	0.49-0.83
		Q4	99	5,805	17.1	0.65	0.51-0.84	0.65	0.50-0.84	0.62	0.47-0.80
		*P_trend_*				<0.001		<0.001		<0.001	
	Yes	Continuous (per SD)	52	3,811	13.6	0.96	0.73-1.27	0.94	0.72-1.22	0.97	0.73-1.30
		*P*				0.779		0.625		0.848	
		Q1	14	800	17.5	1 (ref)		1 (ref)		1 (ref)	
		Q2	13	834	15.6	0.87	0.41-1.84	0.78	0.36-1.67	0.71	0.31-1.61
		Q3	13	1,096	11.9	0.67	0.31-1.42	0.65	0.31-1.40	0.66	0.29-1.51
		Q4	12	1,081	11.1	0.62	0.29-1.34	0.62	0.29-1.35	0.57	0.25-1.29
		*P_trend_*				0.171		0.197		0.182	
Mortality	No	Continuous (per SD)	105	23,477	4.5	0.95	0.79-1.16	0.92	0.76-1.12	0.90	0.73-1.13
(CVD)		*P*				0.635		0.425		0.367	
		Q1	31	5,935	5.2	1 (ref)		1 (ref)		1 (ref)	
		Q2	27	5,938	4.5	0.88	0.53-1.48	0.89	0.53-1.50	0.76	0.45-1.29
		Q3	23	5,800	4.0	0.76	0.44-1.31	0.78	0.45-1.33	0.67	0.38-1.17
		Q4	24	5,805	4.1	0.80	0.47-1.37	0.81	0.47-1.38	0.72	0.41-1.26
		*P_trend_*				0.349		0.362		0.222	
	Yes	Continuous (per SD)	9	3,811	2.4	1.05	0.55-1.98	0.99	0.53-1.85	0.85	0.44-1.66
		*P*				0.886		0.985		0.639	
		Q1	1	800	1.3	1 (ref)		1 (ref)		1 (ref)	
		Q2	1	834	1.2	0.94	0.06-15.03	0.79	0.05-12.79	0.76	0.04-14.93
		Q3	5	1,096	4.6	3.61	0.42-30.89	3.42	0.40-29.60	3.43	0.34-34.24
		Q4	2	1,081	1.9	1.49	0.14-16.44	1.44	0.13-15.97	0.79	0.06-10.55
		*P_trend_*				0.507		0.507		0.848	

^1^ Adjusted for mean total energy intake (2096 kcal for men, 1579 for women) using the residual method. The quartiles values of energy adjusted calcium intake were <458, >458 to <584, >584 to <762 and >762 mg/day for men, and <417, >417 to <529, >529 to <688 and >688 mg/day for women.

^2^ Adjusted for age and sex

^3^ Further adjusted for BMI, PASE, smoking status, alcohol use, education level, self-reported history of diabetes and hypertension, percentage of energy from total fat, percentage of energy from saturated fat

Table 4 shows the unadjusted and adjusted risk estimates of all-cause or CVD mortality by calcium supplemental use. Calcium supplemental use was associated with a significantly reduced risk of all-cause mortality in both unadjusted model and model adjusted for quartiles of dietary energy adjusted calcium intake. The inverse association between calcium supplemental user and all-cause mortality became insignificant when the models were further adjusted for other demographic and lifestyle factors. Similar insignificant inverse trend was observed between CVD mortality and calcium supplemental use in both unadjusted and adjusted models.

**Table 4:** HRs and 95% CIs for all-cause and CVD mortality by use of calcium supplement.

	Calcium supplement use	No. of death	No. of person- years	Rate per 1,000 person years	Crude		Model 1^1^		Model 2^2^		Model 3^3^	
					HR	95% CI	HR	95% CI	HR	95% CI	HR	95% CI
Mortality	No	477	23,477	20.3	1		1		1		1	
(all-cause)	Yes	52	3,811	13.6	0.68	0.51-0.90	0.69	0.52-0.92	0.77	0.58-1.03	0.83	0.62-1.11
	*P*				0.007		0.012		0.077		0.197	
Mortality	No	105	23,477	4.5	1		1		1		1	
(CVD)	Yes	9	3,811	2.4	0.54	0.27-1.06	0.54	0.27-1.07	0.60	0.30-1.18	0.59	0.30-1.18
	*P*				0.071		0.076		0.140		0.138	

^1^ Adjusted for quartiles of dietary energy adjusted calcium intake with mean total energy intake (2096 kcal for men, 1579 for women) using the residual method. The quartiles values of energy adjusted calcium intake were <458, >458 to <584, >584 to <762 and >762 mg/day for men, and <417, >417 to <529, >529 to <688 and >688 mg/day for women.

^2^ Further adjusted for age and sex

^3^ Further adjusted for BMI, PASE, smoking status, alcohol use, education level, self-reported history of diabetes and hypertension, percentage of energy from total fat, percentage of energy from saturated fat

## Discussion

In this prospective cohort study, higher dietary calcium intake was associated with decreased risk of all-cause and CVD mortality in Chinese older people with a mean dietary calcium intake of approximately 600 mg/day. Similar inverse association was observed when the analyses were stratified on calcium supplemental use. Moreover, calcium supplemental use appeared to be associated with reduced risk of mortality, especially all-cause mortality in this population. However, since there was only limited number of deaths, especially CVD deaths among calcium supplemental users, the usefulness of the stratified analysis should be interpreted with cautions. 

Limited studies have examined the association between dietary calcium intake and mortality. These studies were mainly conducted in White population with moderate to high dietary calcium intake. Findings of these studies were however inconclusive. Differences in study design, subjects’ inclusion and exclusion criteria, and covariates in various studies may lead to the mixed findings. While some studies reported reduced risk of death with increasing dietary calcium intake [16,17], other studies reported no association [18,19] or increased mortality risk [4]. 

In a cohort of Swedish men who were free of cancer, CVD and diabetes and did not use dietary supplements at recruitment, dietary calcium was associated with a significantly lower rate of all-cause mortality (HR=0.75, 95% CI=0.63-0.88, *P*_trend_<0.001) and a non-significantly lower risk of CVD death (HR=0.77, 95% CI=0.58-1.01, *P*_trend_=0.064) when the highest intake tertile of dietary calcium (mean=1,953 mg/day) was compared with the lowest (990 mg/day) [17]. In comparison with the studies reporting reduced morality with increasing dietary calcium intake, including our study, data from the EPIC-Heidelberg cohort showed that total dietary calcium intake or calcium supplementation was not associated with CVD mortality. However, the combined effect of dietary calcium intake and supplemental calcium intake was not examined in this study [19]. 

In contrast, recent findings from a Swedish female cohort revealed that women with dietary calcium intake exceeding 1,400 mg/day were associated with higher all-cause and CVD mortality compared with women with lower intakes. More notably, use of calcium tablets was not on average associated with all-cause or cause specific morality, but among women with dietary calcium intake above 1,400 mg/day, use of calcium supplements further increased risk of death in a dose dependent manner [4]. In the National Institutes of Health-AARP Diet and Health Study, both supplemental calcium intake of more than 1,000 mg/day and total calcium intake of 1,500 mg/day and higher were associated with elevated total CVD mortality in men but not in women, whereas dietary calcium intake was not related to total CVD death in either sex [3]. Findings of these studies are likely to suggest that calcium intake should be considered at the issue as a whole, rather than separating it from calcium supplement or dietary intake *per se*. Moreover, the association between total calcium intake and mortality, in particular CVD mortality is likely U-shaped. High calcium intake may be protective in populations with habitual low calcium intake, while it may increase mortality risk in populations with habitual high calcium intake. Therefore, the recommendations regarding calcium supplementation should take into account individual populations. In our study, increasing dietary calcium intake was associated with reduced mortality risk in the multivariate models with adjustment for calcium supplemental use. In addition, similar decreasing risks were observed when the analyses were stratified on calcium supplemental use. Moreover, calcium supplemental use was in general associated with reduced risk of mortality, especially all-cause mortality in our study. Although the usefulness of these results may be questioned by the small number of deaths among calcium supplemental users, our data possibly suggest that calcium supplementation may be protective in populations with habitual low calcium intake while it may increase morality risk in populations with higher calcium intake. This can be further supported by the observations from a meta-analysis that a higher risk of cardiovascular events with calcium supplements was only observed in women with a dietary calcium intake exceeding 800 mg/day and not in women with lower intake levels [6]. 

Serum calcium levels are under tight homeostatic control. Although calcium intake is not normally correlated with serum calcium levels, diets that are too low or too high in calcium may disturb calcium homeostasis, leading to changes in blood levels of calcium or calciotropic hormones. Too low calcium intake may affect mortality or CVD risk possibly through its effect on dyslipidemia, insulin resistance, and blood pressure [2,20]. In contrast, recent evidence suggests that calcium enriched meals can increase serum levels of fibroblast growth factor-23 (FGF23) [21], and higher level of circulating FGF23 are associated with an elevated risk of cardiovascular events and all-cause mortality [22,23]. Furthermore, high calcium intake, in particular intake from calcium supplementation, may exert a harmful effect on cardiovascular health through several plausible mechanisms, such as vascular calcification, induction of a hypercoagulable state, and effects on arterial stiffness [24]. 

The strengths of our study included mortality data retrieved from an official database and adjustment for several potential covariates. However, our study had several limitations. Firstly, dietary calcium intake assessed at baseline may not reflect recent dietary exposure. Secondly, self-reported dietary information collected using FFQ was subject to measurement errors and recall bias. Thirdly, data on the dosage and the frequency of calcium supplemental use were not available, thus the amount of calcium intake from supplementation cannot be quantified. The combined effect of total calcium intake from diet and calcium supplements on mortality risk cannot be investigated. Fourthly, the number of CVD deaths was low and the study may be underpowered to detect any association between dietary calcium intake and CVD mortality, thus a longer follow-up will be required to detect such association. Moreover, although we tried to control for the most important covariates, residual confounding effect may still exist. Finally, although dietary calcium intake did not differ significantly between participants who were included and participants who were excluded for the analysis, there were significant differences in some baseline demographic and lifestyle characteristics between the two groups. In addition, our sample as a whole was of a higher educational standard compared with the general Hong Kong population. Univariate analysis of our data suggested that higher education level was associated with higher dietary calcium intake (data not shown). Therefore, if we were able to include all individuals in the population in the study, the study population could be expected to cover more participants of lower education level and capture a wider range of dietary calcium intake, which in turn a more significant inverse association between dietary calcium intake and mortality risk might be resulted.

## Conclusion

High intake of dietary calcium was associated with reduced risk of all-cause mortality and possibly CVD mortality in Chinese older people with low habitual calcium intake. The association between calcium intake and mortality is likely dependent on the habitual calcium intake. High calcium intake may have a protective effect on mortality in populations, such as Chinese with comparatively low habitual calcium intake, while it may increase mortality risk in populations with high habitual calcium intake. Recommendations regarding calcium supplementation should therefore consider individual population characteristics.
